# Malaria Imported from Ghana by Returning Gold Miners, China, 2013

**DOI:** 10.3201/eid2105.141712

**Published:** 2015-05

**Authors:** Zhongjie Li, Yichao Yang, Ning Xiao, Sheng Zhou, Kangming Lin, Duoquan Wang, Qian Zhang, Weikang Jiang, Mei Li, Xinyu Feng, Jianxin Yu, Xiang Ren, Shengjie Lai, Junling Sun, Zhongliao Fang, Wenbiao Hu, Archie C.A. Clements, Xiaonong Zhou, Hongjie Yu, Weizhong Yang

**Affiliations:** Key Laboratory of Surveillance and Early-warning on Infectious Disease, Chinese Center for Disease Control and Prevention, Beijing, China (Z. Li, S. Zhou, Q. Zhang, J. Yu, X. Ren, S. Lai, J. Sun, H. Yu, W. Yang);; Guangxi Zhuang Autonomous Region Center for Diseases Control and Prevention, Nanning, China (Y. Yang, K. Lin, Z. Fang);; National Institute of Parasitic Diseases, Chinese Centre for Disease Control and Prevention, Shanghai, China (N. Xiao, D. Wang, W. Jiang, M. Li, X. Feng, X. Zhou);; Queensland University of Technology, Brisbane, Queensland, Australia (W. Hu);; Australian National University, Canberra, Australian Capital Territory, Australia (A.C.A. Clements)

**Keywords:** imported malaria, disease outbreak, China, epidemiology, Ghana, parasites, vector-borne infections, Anopheles, Plasmodium, mosquitoes, malaria

## Abstract

During May-August 2013, a malaria outbreak comprising 874 persons in Shanglin County, China, was detected among 4,052 persons returning from overseas. Ghana was the predominant destination country, and 92.3% of malarial infections occurred in gold miners. Preventive measures should be enhanced for persons in high-risk occupations traveling to malaria-endemic countries.

Malaria is a potentially deadly disease caused by infection with *Plasmodium* spp. parasites, which are transmitted to humans through bites from infected *Anopheles* spp. mosquitoes. As part of global malaria elimination actions by the World Health Organization, in 2010, the government of China initiated the National Action Plan for Malaria Elimination to eliminate malaria by 2020 (*1*). In recent years, the incidence of malaria in China has decreased sharply to 0.18 cases per 100,000 persons in 2012 (*2*). However, imported malaria among persons returning from overseas malaria-endemic regions has been documented in some areas of China (*3*,*4*). These imported cases present a new challenge to malaria elimination in China. To facilitate formulation of more effective prevention and control measures for imported malaria at a time of rapidly increasing globalization, we describe the epidemiologic characteristics of a large outbreak of imported malaria among Chinese workers returning from overseas countries, in Shanglin County, Guangxi Zhuang Autonomous Region, in 2013.

## The Study

In Shanglin County, since 2006, >10,000 inhabitants have traveled abroad to conduct gold mining work, most of them to Ghana. In late April 2013, the government of Ghana began to strictly regulate the gold mining industry, which forced many gold miners to return to Shanglin County within a short time. In recent years, no locally acquired malaria cases had been reported in Shanglin County; only sporadic cases of imported malaria had been reported. Because Ghana is hyperendemic for malaria, Shanglin County conducted active malaria screening during May 1–August 31, 2013 among 3 groups: 1) persons with an overseas travel history during the previous year, 2) febrile patients visiting hospitals who had no overseas travel history, and 3) asymptomatic local residents who had no overseas travel history but lived in the same household as persons who had malaria.

All persons who had *P. falciparum* infection were treated with artemisinin-based combination therapy; persons who had no glucose-6-phosphate dehydrogenase deficiency and who were infected with *P. vivax* or *P. ovale* were radically cured with chloroquine combined with primaquine; and persons who had *P. malariae* infection were treated with chloroquine. All persons who had malaria were grouped into inpatients and outpatients, and antimalarial treatments differed according to their clinical situations (Technical Appendix).

Epidemiologic investigation with a standardized questionnaire was conducted. We also collected data on demographic information, history of overseas travel, dates of illness onset and blood sampling, result of blood testing, clinical features, and treatment.

To evaluate the risk for local transmission of malaria, entomologic investigations using light traps and human landing collections methods were conducted in the 4 villages that had a large number of confirmed malaria cases. The numbers of adult *Anopheles* mosquitoes were recorded, and the species of adult *Anopheles* were distinguished.

During the study period, 6,096 persons were tested for *Plasmodium* spp. infections in Shanglin County: 4,052 persons with histories of overseas travel, 1,316 febrile patients visiting local hospitals, and 728 local residents living with persons who had malaria; no one in the 2 latter groups had traveled overseas (Table 1). We detected 874 persons who had malaria, all of whom had traveled overseas. The attack rate was 216/1,000 persons for the persons returning from overseas.

**Table 1:** Results of *Plasmodium* spp. screening by microscopic examination, Shanglin County, China, May 1–August 31, 2013

Items	Overall	Persons with overseas travel	Local febrile patients with no overseas travel	Local residents with no overseas travel living with person with malaria
No. persons screened for malaria	6,096	4,052	1,316	728
No. detected malaria infection	874	874	0	0
Attack rate, %	14.3	21.6	0	0
No. *Plasmodium* species				
* P. falciparum*	827	827	0	0
* P. vivax*	42	42	0	0
* P. malariae*	1	1	0	0
* P. ovale*	1	1	0	0
*Plasmodium* spp. co-infection*	3	3	0	0

*All were *P. falciparum* co-infected with *P. vivax*.

**Figure 1:**
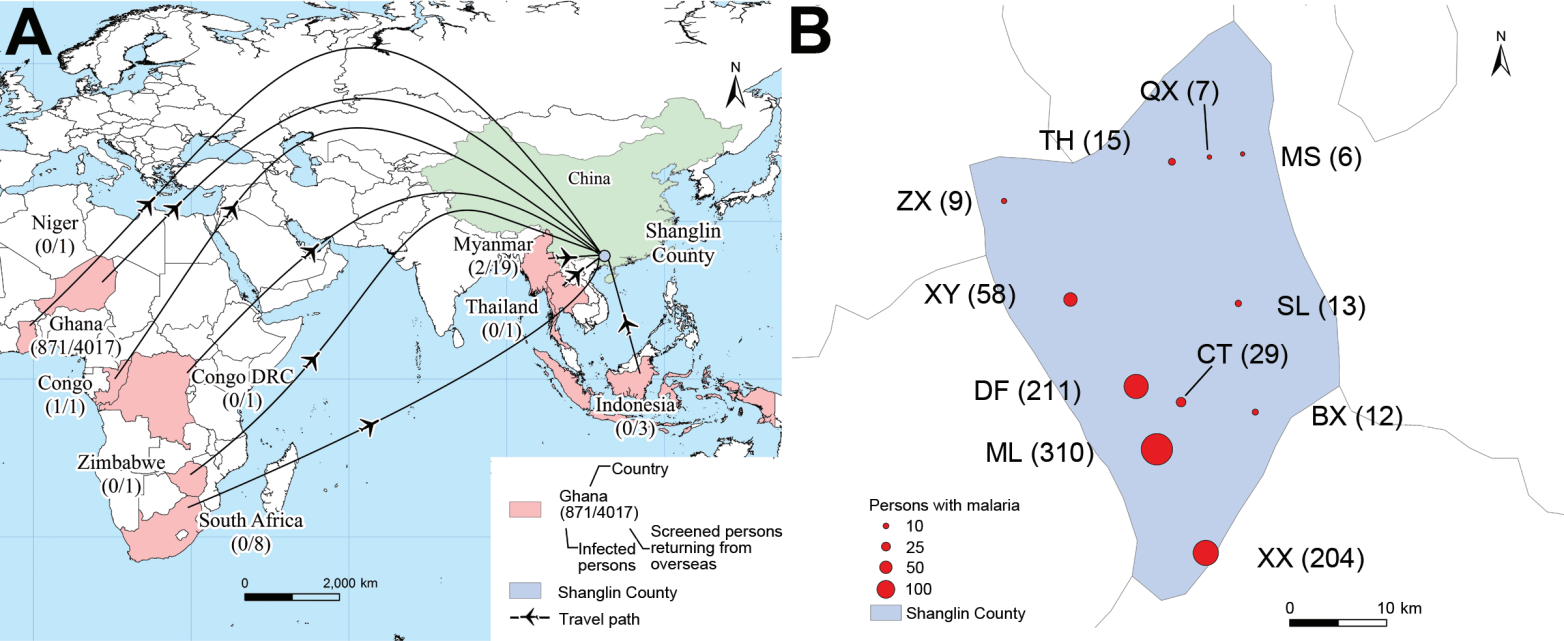
Geographic origin of gold miners returning from oversea (Ghana) and distribution of detected malaria infections, Shanglin County, China, May 1–August 31, 2013. A) Geographic origin of screened miners and persons with malaria. B) Residence of miners who had malaria. DF, Da Feng; ML, Ming Liang; XX, Xiang Xian; CT, Cheng Tai; BX, Bai Xu; SL, San Li; QX, Qiao Xian; MS, Mu Shan; TH, Tang Hong; XY, Xi Yan; ZX-Zhen Xu.

Of the 874 persons who had malaria, 871 (99.7%) had returned from Ghana, 2 from Myanmar, and 1 from the Republic of the Congo (Brazzaville). These persons resided in 11 towns in Shanglin County; most lived in 3 towns: 310 (35.5%) in Mingliang, 211 (24.1%) in Dafeng, and 204 (23.3%) in Xiangxian (Figure 1).

Most persons who had malaria were infected with *P. falciparium* (827 [94.6%]). *P. vivax* was responsible for 42 (4.8%) cases; *P. malariae* and *P. ovale* accounted for 1 case each. Three persons were co-infected with different *Plasmodium* spp. (Table 1).

**Figure 2:**
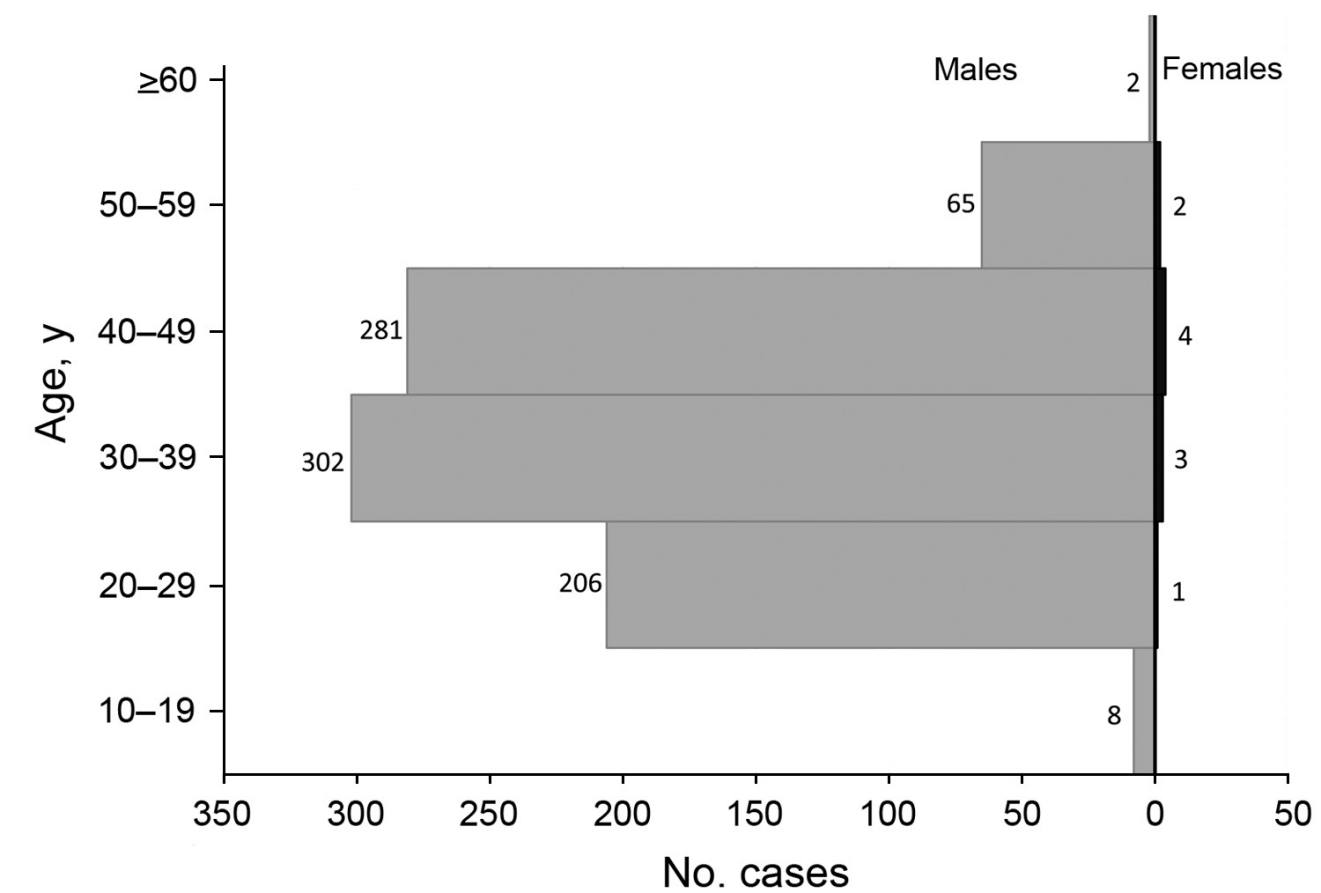
Age and sex of persons who had malaria, Shanglin County, China, May 1–August 31, 2013.

A total of 807 (92.3%) infected persons were gold miners. Nearly all (864 [98.9%]) infected persons were males. Mean age was 36.7 years (range 18–64 years), and most (797 [91.2%]) persons were 20–49 years of age (Figure 2).

A total of 301 (34.4%) *Plasmodium*-positive persons had asymptomatic infections. No deaths occurred. The median interval between return date and diagnosis date was 8 days (range 0–28 days; interquartile range 4–18 days). Among the 369 (42.2%) persons hospitalized for medical treatment, fever >37.3°C (366 [99.2%]), headache (288 [78.0%]), and chills (271 [73.4%]) were the most common symptoms. For hospitalized patients, *P. falciparium* was the predominant species (336 [91.1%]), and *P. vivax* was responsible for 28 (7.6%) cases. Fourteen (3.8%) persons had complicated symptoms. About half (50.7%) of the inpatients had mild to moderate anemia, and 21.1% of those were thrombocytopenic (Table 2).

**Table 2:** Clinical manifestation of malaria among hospitalized persons, Shanglin County, China, May 1–August 31, 2013*

Variable	No. cases (%), n = 369
Common signs/symptoms	
Fever, >37.3°C	366 (99.2)
Fever >38°C	222 (60.2)
Headache	288 (78.0)
Chills	271 (73.4)
Fatigue	215 (58.3)
Dizziness/nausea	213 (57.7)
Sweating	65 (17.6)
Diarrhea	8 (2.2)
Complicated symptoms	14 (3.8)
Liver function impairment	7 (1.9)
Acute renal dysfunction	2 (0.5)
Gastrointestinal impairment	2 (0.5)
Coma	2 (0.5)
Hemolysis	2 (0.5)
Cerebral lesion	1 (0.3)
Severe anemia	1 (0.3)
Acidosis	1 (0.3)
Blood test result, reference range	
Hemoglobin <131 g/L, 130–160	187 (50.7)
Platelet count <85 × 10^9^/L, 85–300	78 (21.1)
G6PD deficiency	45 (12.2)

*G6PD, glucose-6-phosphate dehydrogenase.

The median duration of overseas travel was 356 days for persons who had malaria, which did not differ significantly from persons who did not have malaria (median 354 days; p = 0.7709, 2-tailed Wilcoxon test). All persons who had malaria reported no history of malaria before going abroad, and 871 (99.7%) had taken no mosquito preventive measures during their stay overseas.

A total of 593 *Anopheles* mosquitoes were collected in the 4 villages that had large numbers of persons who had malaria. All mosquitoes were identified as *An. sinensis*.

## Conclusions

We report an unusual, large-scale event of imported malaria among gold miners returning from overseas country to China. Reports have suggested that gold panning activities lead to massive environmental changes, diverting rivers and building basins where vectors can easily breed, thereby increasing the risk for malaria and transmission among gold miners (*5*–*7*).

In this outbreak, just over one third (34.4%) of persons who had malaria were asymptomatic. Because asymptomatic carriers with low-level parasitemia can be reservoirs of infection (*8*,*9*), the high proportion of asymptomatic malaria among the returning miners would pose challenges to identifying and treating infection, and transmission interruption in China (*10*).

In this outbreak, no local malaria transmission was identified. A primary reason is that the local predominant anopheline species is *A. sinensis*, which is refractory to *P. falciparium* (*11*,*12*).

One of our study limitations was that chemoprophylaxis and detailed exposure history in Ghana were not well documented because most returning miners lacked knowledge and awareness of malaria. In addition, recall was likely to have been poor, given that the miners had lived and worked overseas for a long time at the time of investigation.

Considering the remarkably increasing volumes of cross-border travel, malaria imported from overseas countries is a new challenge for malaria elimination in China, as illustrated by the outbreak reported here. Measures to prevent mosquito bites and chemoprophylaxis should be addressed among groups at high occupational risk for malaria. Clinicians and public health practitioners should enhance their awareness of malaria infection among groups returning from oversea malaria-endemic areas, regardless of whether they have common symptoms. Additionally, entomologic surveillance should be conducted in areas with high risk for imported malaria to assess the risk for local malaria transmission.

Technical Appendix Case criteria for outpatient and inpatient malaria treatme
