# Supplementary material for: Malaria Imported from Ghana by Returning Gold Miners, China, 2013
Source: Emerg Infect Dis. 2015 May;21(5):864–7. doi: 10.3201/eid2105.141712 (PMC4412230; doi:10.3201/eid2105.141712)
Supplement: Technical Appendix — Case criteria for outpatient and inpatient malaria treatme [file 14-1712-Techapp-s1.pdf]

Article DOI: http://dx.doi.org/10.3201/eid2105.141712

Malaria Imported by Gold Mining Workers Returning from Ghana, China, 2013

Technical Appendix

Case Criteria for Outpatient and Inpatient Malaria Treatment

Persons whose illness met any one of the following criteria were hospitalized for treatment and medical observation:

1. Severe and complicated cases;
2. A density of *Plasmodium* in the blood smear reaching “++” or above, with “++” deNoting that an average of 6–10 *Plasmodium* parasites were detected per scanned visual field;
3. Body temperature >38.5°C;
4. Specific underlying diseases; and
5. Inability to tolerate the side effects of antimalarial medicines.

Patients with malaria that did not meet the above criteria were treated as outpatients with antimalarial medication provided by prescription.
